# Fact construction as a missing layer in legal AI: a practice-grounded framework for evaluating multi-agent legal consultation

**DOI:** 10.3389/frai.2026.1896166

**Published:** 2026-10-20

**Authors:** Hsuan-Lei Shao, Ying-Chu Yu, Sieh-Chuen Huang

**Affiliations:** 1 Graduate Institute of Health and Biotechnology Law, Taipei Medical University, Taipei, Taiwan; 2 College of Law, National Taiwan University, Taipei, Taiwan

**Keywords:** fact construction, human-AI interaction, large language models, legal AI, legal aid, legal consultation, multi-agent systems, responsible AI

## Abstract

Legal AI research often evaluates large language models (LLMs) on legal reasoning tasks under the assumption that relevant facts are already structured and available. This assumption overlooks a prior and practically consequential stage of legal work: fact construction, namely the transformation of incomplete, fragmented, and interactionally disclosed information into coherent factual narratives. This study conceptualizes fact construction as a distinct evaluation layer in legal AI and introduces a practice-grounded framework for assessing it in AI-assisted legal consultation. Rather than proposing a legal advice system or doctrinal rule-matching architecture, we use a role-separated multi-agent workflow consisting of a fact-constrained Litigant Twin, a structured-questioning LegalAider, a NarrativeAgent for factual synthesis, and an Evaluator for factual-alignment assessment. Using 100 de-identified legal clinic consultation records, we evaluate multiple LLMs under rubric-based assessment, comparing generated factual narratives against original human-authored consultation records in terms of factual accuracy, completeness, and consistency. The results show that LLM-based agents can generate usable factual narratives under constrained conditions, particularly when cases are internally consistent, limited to a single dispute, and supported by clear actor-role relationships. However, cross-model evaluator agreement remains moderate, and outlier analysis reveals recurring structural sources of failure, including anonymization-induced referential ambiguity, third-party reporting, multi-episode case complexity, and numerically dense facts. These findings suggest that reliability in AI-assisted legal consultation depends not only on model capability, but also on interaction design, data representation, institutional record-keeping, and human oversight. By treating fact construction as a distinct analytical layer, this article contributes a practice-grounded framework for evaluating responsible LLM integration in legal settings and highlights the need for factual grounding, contestability, and human-supervised review before downstream legal analysis begins.

## 1 Introduction

Large language models (LLMs) have become a major focus of legal AI research. Their emergence builds on a longer tradition of legal informatics, in which computational methods have been used to organize legal texts, manage large document collections, visualize legal information, and support data-driven legal practice (Jenkins, 2008; Waltl et al., 2015; Conrad et al., 2005; Lachmayer and Cyras, 2021; Sharma et al., 2021; Katz and Dolin, 2021). Recent studies have extended this agenda to LLM-based legal document analysis, contract review, legal question answering, semantic annotation, and benchmark-based evaluation of legal reasoning (Šavelka and Ashley, 2023; Sun, 2023; Shaghaghian et al., 2020; Zhang et al., 2023; Elwany et al., 2019; Guha et al., 2023). Legal AI has therefore become an important testing ground for evaluating how far general-purpose language models can perform legal reasoning and legal text-processing tasks in professional, high-stakes, and normatively structured domains.

Yet much of this evaluation begins after the relevant legal materials or factual premises have already been formulated. LegalBench, for example, provides an influential framework for evaluating LLMs across categories such as issue-spotting, rule-recall, rule-application, rule-conclusion, interpretation, and rhetorical understanding (Guha et al., 2023). In these tasks, facts, legal texts, rules, or questions are presented to the model as structured inputs, and the model is evaluated on whether it can classify an issue, recall a rule, apply a rule, interpret a legal text, or reach a legal conclusion. This design is appropriate for evaluating downstream legal reasoning. It tells us much less, however, about the prior stage in which legally relevant facts must be elicited, clarified, stabilized, and narrated before legal qualification begins.

This tendency is not limited to academic benchmarks. It is also reflected in many contemporary legal AI systems. Public-facing legal QA tools often respond directly to short user prompts by identifying possible legal issues or suggesting next steps, even when the factual basis remains unclear or only partially specified. To be sure, some legal aid and guided-interview systems do incorporate follow-up questions, especially in intake, screening, routing, or document assembly settings. However, these systems are generally designed to determine eligibility, collect standardized information, or support service delivery workflows. Much less attention has been paid to fact construction itself as an independent object of evaluation: whether an AI system can recognize factual insufficiency, conduct structured follow-up questioning, preserve uncertainty, and transform fragmented disclosures into a usable factual narrative before legal analysis begins.

The limitation of this shared design assumption is that it leaves an earlier stage of legal work underexamined. This omission matters because legal rules cannot be responsibly applied when the factual basis remains unclear or incomplete. Prior work in evidence scholarship has long recognized that factual accounts are often constructed from incomplete and uncertain information (Anderson et al., 2005). In real consultations, clients often provide partial, ambiguous, or non-linear narratives, and relevant information may emerge only through follow-up questions. We use the term “fact construction” to describe this pre-reasoning process: transforming fragmented client disclosures into a coherent factual narrative while preserving uncertainty and marking missing information.

Legal consultation records provide a useful empirical setting for studying this pre-reasoning process. Unlike benchmark tasks built from already formulated fact patterns, these records capture the written factual accounts produced during early-stage consultation. Although they are not verbatim transcripts of lawyer-client conversations, they allow us to observe how client narratives are condensed, organized, and partially stabilized into written case facts, while still retaining gaps, ambiguities, and unresolved questions. In this sense, they offer a practice-grounded basis for evaluating whether AI systems can support fact construction before legal analysis begins.

To further develop the account of fact construction advanced above, this article uses a previously reported multi-agent legal aid architecture for a different analytical purpose (Yu et al., 2025). Whereas the earlier work introduced the system design and demonstrated the feasibility of role-separated agents for interactive case fact management, the present article reframes the architecture as a practice-grounded simulation and diagnostic framework for studying fact construction in AI-mediated legal consultation. The framework models legal consultation as an interactional process rather than a single-round question-answering task. It uses de-identified legal clinic records to create a fact-constrained Litigant Twin, which has access to the recorded case facts but discloses them only in response to questions. A LegalAider agent, by contrast, begins without access to the full record and must conduct iterative questioning, clarification, and factual organization. The resulting dialogue is then synthesized by a NarrativeAgent into a factual account and assessed by an independent Evaluator. In this design, the main contribution is not a deployable legal advice system, nor a definitive benchmark for legal consultation AI, but a controlled workflow for making factual elicitation, narrative construction, and failure modes observable. Rather than treating facts as already available inputs to legal reasoning, the framework provides a way to examine how LLM-based agents ask questions, handle uncertainty, and construct reviewable factual narratives before the consultation moves toward legal assessment.

## 2 Related work

### 2.1 Legal consultation as question answering in LLM-based legal AI

Recent legal AI research has increasingly examined whether large language models (LLMs) can support legal information access, legal advice, and legal assistance. A growing body of work studies LLMs as systems that respond to users' legal questions, retrieve relevant legal sources, or generate long-form legal answers (Louis et al., 2024; Hagan, 2023). As highlighted in a recent scoping review by Krook et al. (2024), evaluating the quality of these responses is closely connected to access-to-justice concerns. Because many individuals cannot readily obtain professional legal assistance, LLM-based systems are often evaluated in terms of whether they can provide understandable, useful, and legally accurate responses to lay users' questions or problem descriptions. Recent work has similarly emphasized clarifying-question generation and heterogeneous legal-aid user needs in LLM-mediated assistance, reinforcing the need to treat factual disclosure as an interactional process rather than a simple input for legal reasoning (Zhang et al., 2025; Kuk and Harašta, 2025).

To evaluate whether LLMs can fulfill these practical needs, researchers have adopted this question-answering orientation to test model capabilities. For instance, legal reasoning benchmarks such as LegalBench evaluate whether LLMs can perform tasks such as issue spotting, rule recall, rule application, legal interpretation, and conclusion generation (Guha et al., 2023). Other studies focus more directly on the risks of deploying LLMs in legal information environments, including hallucination, citation errors, unstable responses, and the need for professional supervision and verification (Dahl et al., 2024; Magesh et al., 2025). Together, these studies show that LLM-based legal assistance cannot be assessed only by fluency or general usefulness; factual accuracy, legal grounding, jurisdictional specificity, and reliability remain central concerns.

At the same time, much of this literature operationalizes legal consultation as a static problem of responding to a pre-provided question or problem statement. Even when evaluating a user's “legal problem story,” the primary focus remains on the system's final output: whether it identifies relevant issues, cites appropriate sources, or communicates clearly (Hagan, 2023; Krook et al., 2024). While this orientation is suitable for evaluating information retrieval, it leaves underexamined the critical, prior stage in which a lay user's raw narrative must be elicited, clarified, and reconstructed. Because a system's response is only as good as its factual premise, evaluating LLM-based consultation solely on post-statement output quality provides an incomplete picture. To address this gap, the next subsection turns to lawyering and clinical legal education literature, where legal consultation is fundamentally understood as an interactive, interview-based process of listening, questioning, and fact-identification.

### 2.2 Client interviewing, legal clinics, and factual elicitation

Legal consultation has long been understood in lawyering and clinical legal education literature as more than the delivery of legal information. In civil legal aid standards, the initial exploration of a client's problem requires the practitioner to interview the client, elicit pertinent facts and circumstances, identify legal issues and client objectives, and explain the steps needed to address the problem (American Bar Association, 2021). The same standards emphasize that clients may not organize facts in the manner a lawyer would use for effective presentation of a case, may not perceive the relevance of some facts, and may overestimate the importance of others (American Bar Association, 2021). This account treats consultation as a process of factual discovery and organization, not merely as a request for legal advice followed by a professional answer.

Clinical legal education scholarship develops a similar view. Legal clinics are designed not only to provide assistance to members of the community, but also to train students in practical lawyering skills, including interviewing, taking instructions, fact investigation, legal analysis, counseling, negotiation, and professional responsibility (Quigley, 1995; Du Plessis, 2018). Interviewing is therefore both a service function and a pedagogical practice. In this setting, facts are not simply received as fixed inputs. They are gradually elicited, tested, clarified, and recorded through a structured interaction between the client, the student interviewer, and the supervising legal professional. This literature is particularly important for legal AI because it shows why legal consultation cannot be reduced to question answering.

Legal clinic records provide a useful, though imperfect, empirical trace of this process. Such records stand between the client's initial account and the legally refined narratives that appear in pleadings or judgments. This intermediate status makes legal clinic records especially relevant for studying fact construction in legal AI. They allow evaluation to move beyond final legal answers and to examine whether an AI-mediated consultation workflow can recover, organize, and preserve legally meaningful facts at an early stage of legal assistance.

### 2.3 Multi-agent systems and role separation in legal consultation

Multi-agent systems have long been studied in artificial intelligence as a way to model distributed reasoning, task specialization, coordination, negotiation, and interaction among autonomous agents (Wooldridge and Jennings, 1995; Stone and Veloso, 2000). In legal and normative domains, multi-agent perspectives have also been used to model institutional roles, rule-governed interaction, argumentation, and normative coordination (Boella et al., 2006; Rahwan and Simari, 2009). While these early frameworks relied primarily on symbolic AI, deontic logic, and formal argumentation trees, the advent of large language models (LLMs) introduces a paradigm shift. Rather than treating an LLM as a single text generator, agent-based approaches assign models to roles, equip them with task instructions or tools, and organize their interactions within a workflow. In the legal domain, recent surveys report that LLM-based legal agents have transitioned from single-agent setups to complex multi-agent simulations, applied to tasks such as legal information retrieval, legal question answering, judgment prediction, and legal text generation, while also identifying multi-agent coordination, trustworthiness, and dynamic evaluation as continuing challenges (Yang et al., 2025).

This development is relevant to legal consultation because consultation is not a single cognitive act. It involves multiple functional activities: a client discloses information, a legal professional asks follow-up questions, factual details are clarified and organized, and the resulting account is reviewed for completeness and consistency. A single-agent chatbot can merge these activities into one conversational interface, but doing so turns the process into a black box. When the same model elicits, interprets, summarizes, and evaluates facts simultaneously, a failure in the final output provides limited diagnostic information about whether the error arose from insufficient disclosure, poor questioning, flawed synthesis, unsupported legal inference, or weak evaluation. Role separation therefore has methodological value: it decomposes consultation into observable stages and allows each component to be constrained and examined more precisely.

Recent legal AI studies illustrate different uses of multi-agent design. Legal-agent benchmarks and rubric-based LLM evaluation further support the need to assess intermediate processes and to treat evaluator scores as diagnostic rather than definitive measures (Li et al., 2025; Siro et al., 2026). Some work uses multi-agent debate to improve legal judgment prediction, assigning agents to dynamic adversarial or evaluative roles in order to approximate the dialectical nature of courtroom reasoning (Chen et al., 2025). Other work develops interactive legal environments for LLM-based agents, emphasizing that real legal practice is dynamic, multi-turn, and procedurally constrained rather than merely a matter of static benchmark performance (Jia et al., 2025). More directly related to legal aid, prior work has proposed a multi-agent framework for case consultations in which specialized agents emulate iterative questioning, clarification, and synthesis processes, using real-world legal aid case records rather than synthetic fact patterns (Yu et al., 2025). These studies suggest that multi-agent design can be used not only to improve model performance, but also to represent the procedural structure of legal work.

Building upon these insights, a role-separated design becomes indispensable when the object of evaluation extends beyond the final answer to the process of information elicitation itself. In legal consultation, factual errors can be introduced at different stages: a client-simulation component may fail to disclose critical constraints; a questioning component may omit necessary follow-up prompts; a synthesis component may distort facts; or an evaluation component may apply rubrics inconsistently. By explicitly separating these functions, the entire consultation workflow becomes traceable, enabling researchers to pinpoint precisely where fact construction succeeds or breaks down.

### 2.4 Evaluation, factual grounding, and LLM-as-judge

Evaluation is a central challenge for legal AI because many legally meaningful outputs are open-ended. Legal answers, consultation notes, factual narratives, and legal summaries often cannot be assessed by exact match against a single correct string. Unlike classification tasks, these outputs may vary in wording, ordering, level of detail, and communicative style while still preserving the same legally relevant information. Legal AI benchmarks such as LegalBench have advanced the evaluation of legal reasoning by organizing tasks around capabilities such as issue spotting, rule recall, rule application, interpretation, and conclusion generation (Guha et al., 2023). However, open-ended consultation outputs require additional forms of assessment because the relevant question is not only whether the system reaches a legally plausible conclusion, but also whether its narrative remains faithful to the facts available in the source material.

This concern connects legal AI evaluation to broader work on factual grounding. Factual grounding asks whether a generated response is supported by a specified context or source document, rather than merely whether it is fluent or plausible. The FACTS Grounding benchmark, for example, evaluates whether long-form model responses are factually accurate with respect to a provided document and uses multiple frontier LLM judges to assess grounding while checking agreement with human raters (Jacovi et al., 2025). This approach is relevant to legal consultation because a factual narrative generated from a consultation record should be evaluated against the record itself. In this setting, hallucination is not limited to inventing legal rules or citations. It also includes adding unsupported actors, dates, transactions, causal relationships, procedural events, or intentions that are not grounded in the consultation record.

Identifying such nuanced factual discrepancies at scale, however, presents a significant methodological hurdle. Recent work has increasingly turned to LLMs as evaluators to address this scalability challenge. General LLM-as-judge research suggests that strong models can approximate human preferences in some open-ended evaluation settings, while also warning that such judgments may be affected by position bias, verbosity bias, self-enhancement bias, and limited reasoning ability (Zheng et al., 2023). Recent surveys similarly emphasize that reliability, consistency, bias mitigation, and domain adaptation remain central challenges for building trustworthy LLM-as-judge systems (Gu et al., 2024). These concerns are particularly significant in legal domains, where an evaluator must look beyond surface-level fluency to scrutinize the multi-layered nature of legal texts.

Legal-domain evaluation therefore requires heightened caution in using LLM-based judges. Specifically, prior work on legal question answering has proposed separating factual accuracy from reasoning quality, reflecting the possibility that an answer may rely on correct facts but unsound reasoning, or conversely may present plausible reasoning while misstating the relevant facts (Gultekin et al., 2025). Work on legal summarization has also shown that different stakeholders may evaluate the same legal output differently, depending on whether they value doctrinal precision, procedural detail, accessibility, or task-specific usefulness (Pang et al., 2025). These studies suggest that legal AI evaluation should not rely on a single undifferentiated quality score, but rather on disentangled, multi-dimensional metrics. In the present study, we adopt this principle at the level of task separation: factual construction is evaluated separately from downstream legal reasoning, doctrinal classification, legal advice quality, and outcome prediction.

The present study adopts this caution by treating the Evaluator not as an authoritative legal decision-maker or a validated measurement tool, but as a controlled diagnostic instrument. The evaluation target is deliberately narrow: factual alignment between the generated narrative and the original consultation record. The Evaluator applies fixed criteria of accuracy, completeness, and consistency, and its scores are interpreted as diagnostic signals rather than final judgments of legal usefulness or benchmark validity. Model-driven evaluation can help identify patterns across cases and generator models, but its validity requires further human calibration and is not resolved in this article.

## 3 Materials and methods

Before describing the workflow, it is important to distinguish between a legal consultation record and a factual narrative. In this study, a legal consultation record refers to a human-authored institutional record produced in the context of legal clinic practice. It is not a verbatim transcript of the client's account, nor is it an adjudicated finding of fact. Rather, it is an intermediate record prepared from the consultation process and contains, in structured form, the factual background, the client's questions, and, in the original form, proposed legal responses. For the experiment, only the factual background and client-question fields were used as source materials; the proposed legal response field was excluded.

By contrast, the factual narrative is the model-generated reconstruction produced after the simulated consultation interaction. It is intended to synthesize the disclosed facts into a coherent, neutral account without providing legal conclusions, doctrinal classification, or legal advice. The evaluation therefore compares the generated factual narrative with the human-authored consultation record as a practice-based reference account. This reference account should not be understood as raw client testimony or an independently verified factual record, but as the closest available institutional record of the factual information collected in real legal consultation practice.

### 3.1 Task definition: fact construction before doctrinal legal analysis

The task evaluated in this study is legal fact construction. Given an original de-identified legal consultation record, the framework simulates a structured consultation interaction and produces a factual narrative. The generated narrative is then compared with the original human-authored consultation record as a reference account. The evaluation asks whether the generated narrative preserves the factual content available in that record, avoids unsupported additions, and maintains internal consistency. The original record is not treated as a definitive statement of legal truth, but as the practice-based reference against which factual alignment can be assessed.

The task deliberately excludes doctrinal legal analysis. The framework does not determine causes of action, legal claims, defenses, remedies, applicable legal rules, or likely litigation outcomes. This exclusion defines the scope of the experiment. The study asks whether an AI-mediated consultation workflow can reconstruct a usable factual account before legal analysis begins. A factual narrative that omits actors, dates, transactions, procedural history, or numerical details may distort later legal analysis, even if the subsequent reasoning model is otherwise capable. Factual adherence is therefore treated as a necessary, though not sufficient, condition for reliable legal AI assistance.

At the same time, factual adherence is not identical to legal relevance. Early-stage consultation records may include background information, emotional descriptions, and details whose legal significance is uncertain or marginal. A mature legal summary may eventually need to filter, prioritize, or reorganize such material in light of legal issues. In this study, however, factual adherence is treated as the first evaluation layer because it establishes whether the system can preserve the information made available during intake. Relevance filtering, issue selection, and doctrinal classification are therefore left to subsequent layers of analysis and future work.

### 3.2 Practice-grounded multi-agent framework

The framework decomposes legal consultation into four agent roles: Litigant Twin, LegalAider, NarrativeAgent, and Evaluator. Figure 1 presents the overall workflow. The purpose of this role separation is methodological. It makes factual disclosure, elicitation, narrative synthesis, and evaluation separately observable, rather than treating consultation as a single undifferentiated chatbot response.

**Figure 1:**
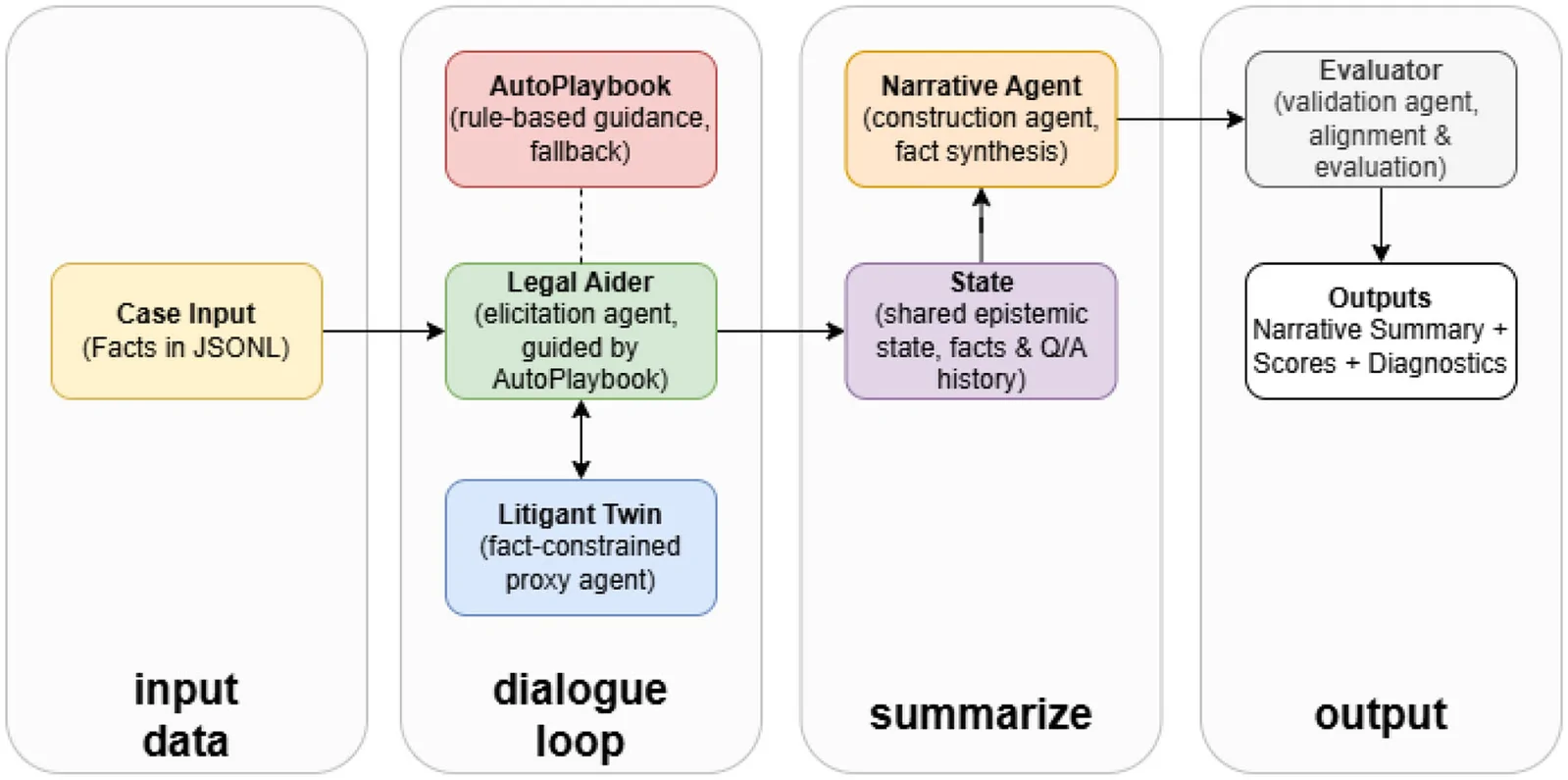
Overview of the multi-agent framework. A LegalAider queries a fact-constrained Litigant Twin, whose responses are synthesized by a NarrativeAgent. An independent evaluator assigns continuous scores and diagnostic observations based on factual alignment.

The **Litigant Twin** is a fact-constrained proxy agent. It receives the original case record as its factual source and is instructed to answer questions only on the basis of the recorded facts. If a requested detail is absent, unclear, or uncertain in the source record, it must indicate that limitation rather than invent information. The design draws on user simulation ideas in task-oriented dialogue evaluation, but it is not a full digital twin in the technical sense. It does not seek to model the litigant's personality, memory, preferences, or strategic behavior. Instead, it functions as a controlled proxy for simulating gradual factual disclosure in legal consultation (Sekulić et al., 2024). Figure 2 illustrates this design principle. The component name “Litigant Twin” is retained as part of the system architecture. However, the underlying records concern legal clinic clients seeking consultation, many of whom had not initiated litigation. Accordingly, except when referring to the agent itself, this article uses “client” to describe the person seeking legal consultation.

**Figure 2:**
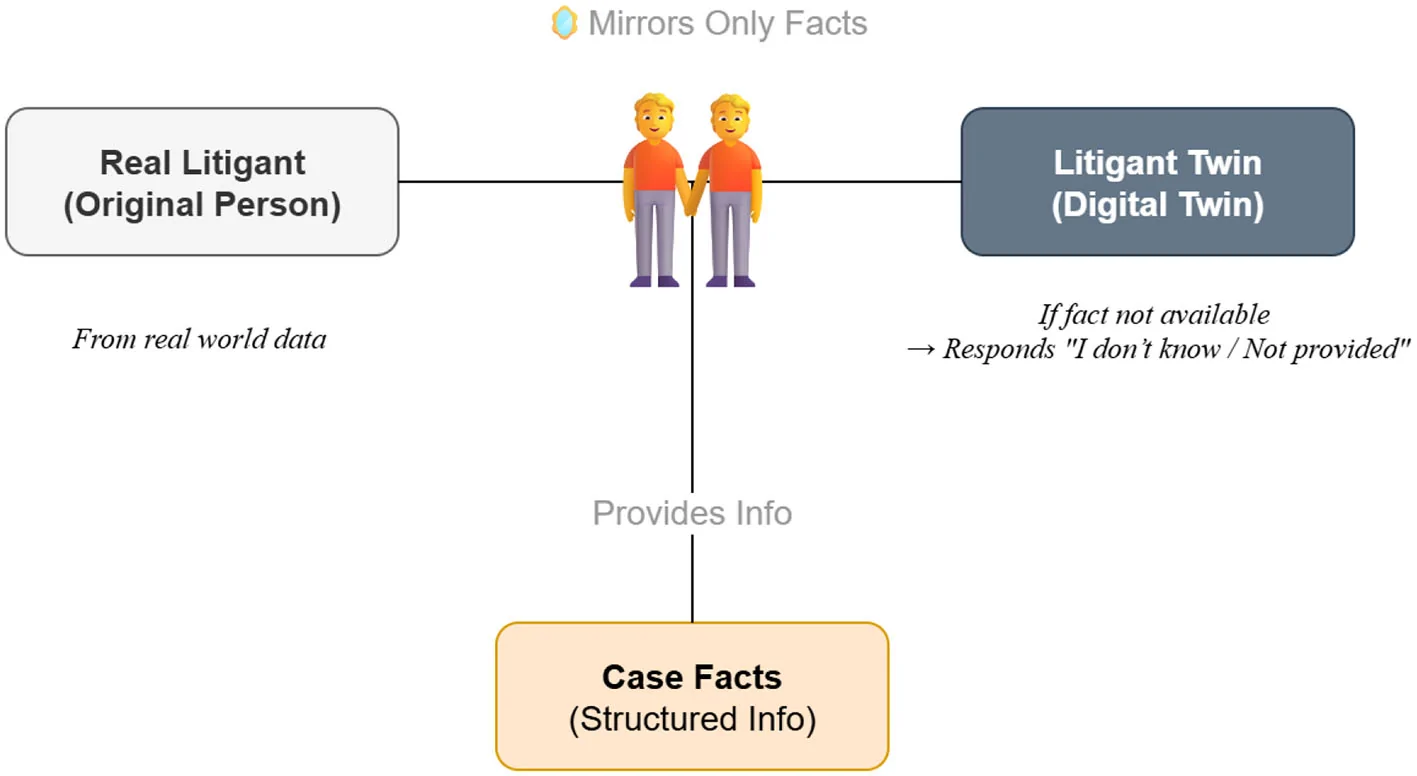
Fact-constrained design of the Litigant Twin. The proxy agent represents the litigant only through recorded case facts and returns explicit non-responses when information is unavailable.

The **LegalAider** is an elicitation agent. Its task is not to provide legal advice or conduct doctrinal legal analysis, but to ask structured, non-redundant questions that clarify the factual narrative. It seeks to identify actors, timelines, events, transactions, injuries, procedural developments, and other information that may be necessary for later human legal review.

The **NarrativeAgent** is a factual synthesis agent. It transforms the information disclosed during the simulated consultation into a coherent factual narrative. The NarrativeAgent is instructed to remain within the disclosed factual record, avoid legal conclusions, and produce a narrative suitable for review by a legal professional or clinic supervisor.

The **Evaluator** is a validation agent. It compares the generated factual narrative with the original human-authored consultation record under a fixed rubric. The rubric focuses on factual accuracy, completeness, and consistency. It also identifies omissions, contradictions, unsupported additions, and areas of uncertainty. The Evaluator may be instantiated using a different LLM from the NarrativeAgent as a methodological precaution against style-based self-preference, although this design does not eliminate the need to examine evaluator-specific scoring tendencies.

### 3.3 Data source and preprocessing

The dataset consists of 100 real-world legal consultation case records provided by the Legal Service Society at National Taiwan University. The records were created during the 2021–2023 academic years, with consultation dates ranging from September 2021 to May 2024. They were maintained as Chinese electronic consultation forms. Each record contains administrative information, case-status information, a student-prepared factual account, questions raised by the client, and a proposed legal response. These records are not verbatim transcripts of lawyer–client conversations. Rather, they are written consultation records prepared in the course of clinical legal service, documenting the facts presented during consultation and the questions that the consultation seeker wished to raise.

For the present experiment, the factual source provided to the Litigant Twin was limited to two fields in each record: the case facts field and the client's questions field. The case facts field served as the primary factual source because it contains the student-prepared account of the factual background disclosed during consultation. The client's questions field was used as an additional source because it preserves the concerns and problem orientation expressed by the person seeking consultation. Administrative fields, including the date, case number, group number, recorder, case status, case progress, and court docket information, were used only for internal organization, dataset description, or de-identification review. The proposed answer field, which contains legal analysis, legal advice, and references to legal rules, was excluded from the input. This exclusion was necessary to maintain the conceptual boundary of the experiment: the framework evaluates fact construction before legal analysis, not the model's ability to reproduce or apply legal advice already prepared by human consultants.

The 100 cases were selected from the available records on the basis of factual completeness. Because the purpose of this study is to evaluate factual elicitation and narrative reconstruction, records were included only when the case facts field contained enough information to support meaningful comparison between the original record and the generated factual narrative. The resulting dataset covers common legal aid categories, including consumer disputes, family matters, debt and labor disputes, traffic accidents, criminal matters, property disputes, and tort liability claims. The sample should therefore be understood as a criterion-based research dataset rather than a random sample of all legal clinic consultations.

Before analysis, all records were de-identified. Personal names, phone numbers, addresses, institutional identifiers, court case numbers, and other potentially identifying details were removed or replaced with neutral placeholders such as “Party A,” “Party B,” or “Company X.” The records were then converted into a machine-readable format for multi-agent processing. This preprocessing was necessary to protect privacy, but it also became a substantive source of evaluation difficulty. As shown in the outlier analysis, anonymized placeholders sometimes disrupted role consistency and referential grounding during interaction.

The use of real consultation records differentiates this study from synthetic legal dialogue experiments. At the same time, these records should not be treated as raw client narratives or as fully adjudicated factual findings. They are intermediate factual products produced in a clinical legal setting: condensed, partially organized, and often incomplete accounts of legally relevant events. They retain features common in practice, including incomplete disclosure, heterogeneous narrative structures, multiple actors, overlapping legal and non-legal concerns, and variable levels of detail. These characteristics make the dataset particularly informative for examining how fact construction operates as an interactional and institutionally embedded process, rather than as a simple summarization task.

### 3.4 Experimental design and model assignment

The experiment uses a role-separated multi-agent workflow. For purposes of model assignment, however, the Litigant Twin, LegalAider, and NarrativeAgent were treated as a single Generator. Within each Generator condition, the same model was used for all three agents. For each of the 100 cases, the de-identified consultation record was first provided as a constrained factual source to the Litigant Twin. The Litigant Twin disclosed information only through simulated interaction. The LegalAider then conducted multi-turn questioning, and the NarrativeAgent generated a factual narrative based on the resulting dialogue. Finally, the Evaluator compared the generated narrative with the original human-authored consultation record.

Multiple widely used general-purpose LLMs were instantiated in the Generator and Evaluator roles. In the reported evaluator–generator matrix, model names are abbreviated as GPT-4o, GPT-4o-mini, and Claude-3.5-Haiku. For each generator condition, the same model was used for the Litigant Twin, LegalAider, and NarrativeAgent. The Evaluator was then varied independently. This produced a 3 × 3 evaluator–generator design over 100 case records, yielding up to 900 possible evaluator–generator–case outputs before exclusion of missing or invalid evaluator outputs. The design enables comparison across generator workflows and evaluator models while holding the input records, workflow structure, and role-specific prompt templates constant.

This design choice has an important interpretive implication. Differences across Generator conditions should be understood as differences in the performance of the entire fact-construction workflow, not as isolated differences in narrative synthesis alone. A higher or lower score may reflect variation in simulated client responses, questioning strategy, dialogue development, or final narrative generation. This is appropriate for the purpose of the present study, which evaluates fact construction as an interactional process rather than as a single-step summarization task. However, the design does not allow separate causal attribution to the Litigant Twin, LegalAider, or NarrativeAgent components.

The experiment was conducted using fixed prompt templates and controlled generation settings. The prompts specified the agent role, the permissible information source, the required output format, and the evaluation criteria. In particular, the Litigant Twin was instructed not to introduce facts absent from the source record; the LegalAider was instructed to conduct structured, non-redundant follow-up questioning; the NarrativeAgent was instructed to produce a factual narrative rather than legal analysis; and the Evaluator was instructed to assess factual alignment rather than legal correctness. This design preserves the conceptual boundary of the study: the experiment evaluates fact construction before legal analysis, rather than end-to-end legal advice generation.

### 3.5 Evaluation criteria

The scoring rubric guides the Evaluator along three dimensions. First, accuracy asks whether the generated narrative avoids contradictions, hallucinated facts, and unsupported additions. Second, completeness asks whether important factual elements from the original record are preserved, including actors, events, timelines, transactions, injuries, procedural developments, and numerical details where relevant. Third, consistency asks whether the narrative represents actors, events, dates, transactions, and causal relationships in a coherent and non-conflicting manner. These dimensions are used to guide the overall similarity score rather than to produce separate sub-scores. The representative prompt templates, factual-alignment evaluation rubric, and output schemas are provided in the Supplementary material.

The resulting score should therefore be understood as a composite factual-alignment index guided by the three rubric dimensions of accuracy, completeness, and consistency, rather than as three independently validated sub-scores. Accuracy refers to whether the generated narrative preserves the factual content of the reference record without unsupported additions; completeness refers to whether key factual elements are retained; and consistency refers to whether the generated narrative avoids internal contradictions and conflicts with the reference record.

This model-driven scoring approach is used for scalability and reproducibility, but it is not treated as a substitute for human legal judgment. The scores are interpreted as comparative diagnostic signals generated under fixed criteria. They are therefore used to compare patterns across generator and evaluator models, not to establish definitive judgments about the legal adequacy of any individual narrative.

To improve interpretability, aggregate scores are supplemented by cross-evaluator comparison and qualitative outlier analysis. In the outlier analysis, cases from the low-scoring or unstable portions of the score distributions were examined by comparing the generated narratives with the original records and the Evaluator's diagnostic comments. This analysis was used to identify recurring structural sources of mismatch, including omissions, unsupported additions, role confusion, incomplete disclosure, and failures to preserve timelines or numerical details.

### 3.6 Statistical analysis

As an exploratory diagnostic analysis of the LLM-as-a-judge scores, we summarized mean similarity scores, standard deviations, and valid sample sizes across evaluator–generator combinations. Because each case was scored under multiple model conditions, we treated case ID as the repeated unit. We conducted Friedman tests on case-aggregated scores to examine generator-level and evaluator-level variation. These analyses were interpreted as exploratory and diagnostic rather than as a formal benchmark or definitive model ranking.

## 4 Results

### 4.1 Overall performance and evaluation stability

Across the 100 case records, the multi-agent workflow generally produced structured factual narratives that remained within the information disclosed during the simulated consultation. Higher scores were more often observed in cases with internally consistent records, a single dispute structure, and clear actor-role mappings. Lower and more variable scores appeared in cases involving multiple episodes, ambiguous anonymized referents, third-party reporting, or dense numerical information, as examined in the outlier analysis below.

Table 1 reports the evaluator–generator score matrix. Rows indicate the model used as the Evaluator, and columns indicate the model used as the Generator. Each cell reports the mean similarity score, standard deviation, and valid sample size.

**Table 1:** Evaluation × generation performance: mean similarity score, standard deviation, and sample size.

Evaluator \ generator ave. (SD, *n*)	GPT-4o	GPT-4o-mini	Claude-3.5-Haiku
GPT-4o	0.74 (0.15, 97)	0.75 (0.15, 100)	0.79 (0.13, 92)
GPT-4o-mini	0.72 (0.12, 100)	0.70 (0.14, 100)	0.77 (0.12, 100)
Claude-3.5-Haiku	0.86 (0.07, 100)	0.85 (0.08, 100)	0.88 (0.08, 100)

Values are mean similarity scores, with standard deviations and valid sample sizes reported in parentheses. Missing or invalid evaluator outputs were excluded from the summaries.

After excluding missing or invalid evaluator outputs, 889 valid scores were retained for the descriptive summaries. For the repeated-measures tests, we used the 89 cases with complete valid scores across all evaluator–generator cells. A Friedman test on case-aggregated generator scores suggested a generator effect, $\chi_{\left(2\right)}^{2}=50.46$, *p* < 0.001. A parallel Friedman test on case-aggregated evaluator scores showed a stronger evaluator effect, $\chi_{\left(2\right)}^{2}=151.02$, *p* < 0.001, indicating that score distributions were substantially shaped by evaluator-specific calibration. Accordingly, these results should be interpreted as diagnostic evidence of evaluator sensitivity and cross-model calibration differences, rather than as a definitive ranking of generator performance.

This finding should be interpreted cautiously. Higher evaluator-assigned scores do not necessarily indicate that the corresponding generator produced objectively superior narratives. They may also reflect evaluator-specific leniency, stricter or broader interpretations of factual alignment, or different sensitivities to omissions and unsupported additions. Prior work on LLM-as-judge has identified risks of position bias, verbosity bias, and self-preference bias (Zheng et al., 2023; Ye et al., 2024; Wataoka et al., 2024). In the present dataset, we did not observe a clear aggregate pattern in which a model systematically assigned the highest scores to its own generated narratives. However, this result should not be understood as evidence that self-favoring behavior is absent. Rather, within this constrained factual-alignment task, evaluator-specific scoring tendencies appear more salient than generator-specific self-preference.

### 4.2 Cross-evaluator consistency

Figure 3 reports Spearman rank correlations between evaluator models under different generation conditions. The correlations are positive but moderate, ranging from 0.54 to 0.77 across the reported conditions. This indicates that evaluator models share some ranking tendencies, but they do not order the generated narratives in the same way. The result suggests that model-driven evaluation should not rely on a single evaluator model when used in legal settings. Multiple evaluators, human calibration, or targeted human audit are needed to interpret score differences more reliably.

**Figure 3:**
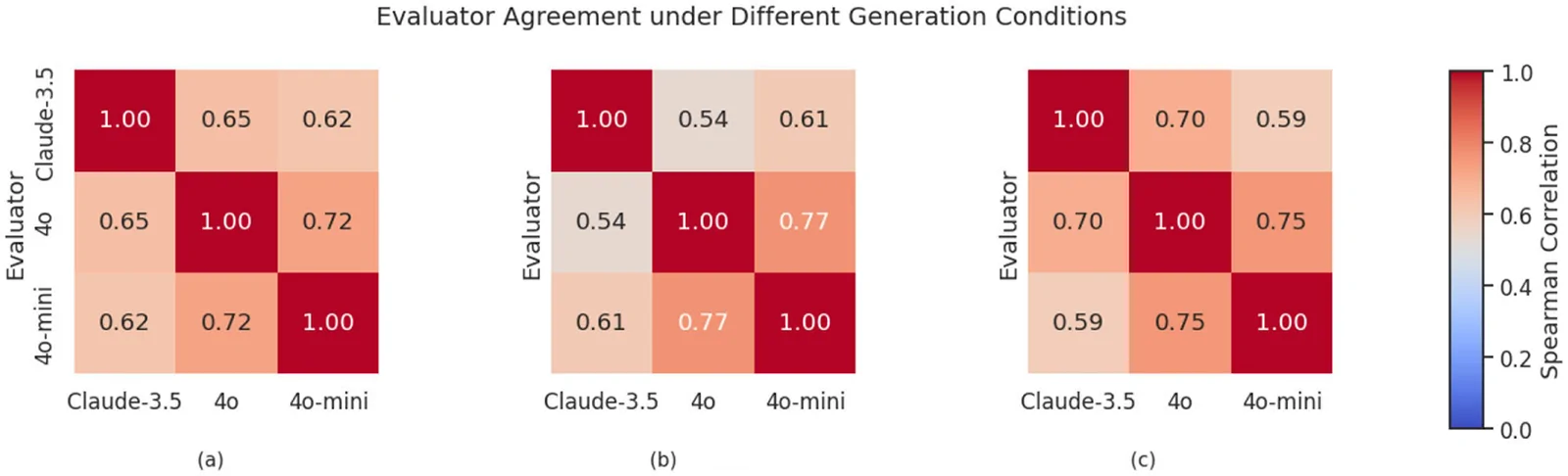
Spearman rank correlations between evaluator models under different generation conditions. **(a)** 4o. **(b)** 4o-mini. **(c)** Claude.

Figure 4 shows the distribution of similarity scores across evaluator–generator combinations. Most generated narratives fall within a middle-to-high scoring range, while a smaller number of cases form a long tail of low or unstable scores. These long-tail cases are analytically important because they reveal conditions under which the fact-construction workflow becomes fragile.

**Figure 4:**
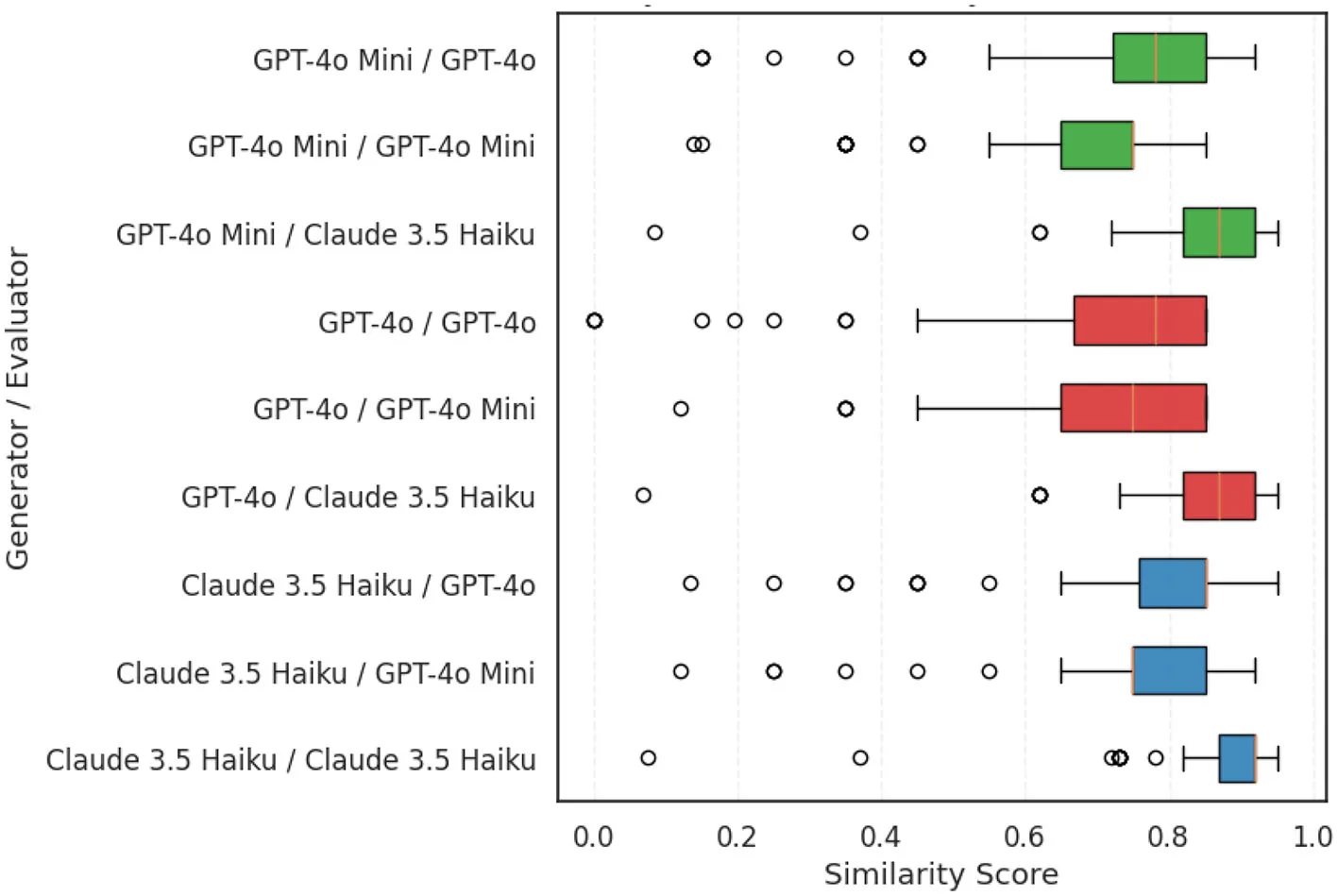
Distribution of similarity scores across evaluator–generator combinations.

### 4.3 Qualitative interpretation of scores

Table 2 illustrates how numerical scores were interpreted through qualitative review. High scores generally correspond to narratives that preserve the main actors, events, and causal sequence of the original record. Mid-range scores require more careful interpretation. They may reflect genuine omissions or weak narrative structure, but they may also reflect evaluator strictness toward missing details that do not substantially alter the core factual account. Low scores typically indicate substantial divergence from the original record or failure to recover the structure of the dispute.

**Table 2:** Representative cases illustrating qualitative interpretation of evaluation scores.

Case	Case type	Score	Analytical positioning
A	Consumer	0.92	High score: author review confirms that the narrative is almost fully aligned with the original facts.
B	Labor	0.62	Mid-range score: author review suggests that the score is conservative because the narrative preserves the core factual account despite missing some details.
C	Fraud	0.62	Mid-range score: author review supports the score because omissions and weak narrative structure affect factual alignment.
D	Mental health-related	0.30	Low score: author review confirms substantial divergence from the original factual structure.

This qualitative interpretation is necessary because the numerical score alone does not reveal whether disagreement arises from generator failure, evaluator strictness, ambiguity in the source record, or institutional preprocessing. The same numerical score may therefore correspond to different diagnostic situations. For this reason, the study treats scores as entry points for diagnostic analysis rather than as final measures of legal usefulness.

### 4.4 Outlier and failure mode analysis

Outliers were identified from the long-tail portions of the score distributions and from cases with unstable evaluations across evaluator models. The purpose of this analysis was not merely to identify poorly performing cases, but to examine the structural conditions under which the fact-construction workflow became unreliable. For these cases, we compared the generated narrative against the original record and examined evaluator comments. Table 3 summarizes the main failure categories.

**Table 3:** Outlier analysis by qualitative category.

Category	Case ID	Notes
*Anonymized referents and role confusion*	105	Anonymized placeholders and third-person phrasing caused referential confusion, leading to ineffective follow-up and sparse summaries.
	040	Role identifiers in legal aid records could not be consistently resolved during dialogue, resulting in missing contractual and statutory details.
*Third-party narration*	105	The consultation was reported by a friend rather than the involved party, limiting access to key factual details.
	136	The inquirer described events involving a partner, repeatedly deferring details and preventing factual completion.
*Multi-episode and multi-timeline case complexity*	121	Multiple incidents and legal proceedings were reduced to a single superficial episode in the generated summary.
	079	Subsequent reporting, medical examination, and procedural developments were omitted during interaction.
	113	Critical timelines and asset details were not articulated, yielding an overly generic narrative.
	144	Key actors and legally salient steps were missing, obscuring the structure of the dispute.
*Information-dense numerical sensitivity*	164	The summary omitted transactional steps despite clear monetary records, amplifying evaluator disagreement.
	163	Evaluators differed in sensitivity to numerical breakdowns and compensation calculations, producing divergent scores.

Four patterns emerge. First, anonymized role labels can destabilize the mapping between actors and their legal or factual roles. When records replace names with A/B/C or similar placeholders, the agents may fail to maintain stable referents across turns. This problem is especially serious when the same case involves multiple family members, contractual parties, intermediaries, or institutional actors. In such cases, the LegalAider may ask formally relevant questions but direct them toward an unstable or misidentified actor, and the NarrativeAgent may then produce a sparse or internally confused factual account.

Second, third-party narration introduces informational distance between the legal clinic client and the legally relevant events. In cases where the client reports events involving a partner, friend, or family member rather than events directly experienced by the client, the Litigant Twin may repeatedly indicate that key details are unknown or unavailable. This behavior is faithful to the fact-constrained design, but it also prevents the consultation from reconstructing the full factual chain. The resulting narrative may therefore over-preserve uncertainty even when the original human-authored record contains a more complete account.

Third, multi-episode and multi-timeline cases are difficult when the interaction does not first separate distinct events, dates, and procedural developments. Some records combine several incidents, later reports, medical examinations, administrative steps, criminal proceedings, or settlement discussions. If the LegalAider does not establish a timeline at the beginning of the dialogue, the later synthesis tends to collapse heterogeneous episodes into a single simplified narrative. This produces summaries that may appear coherent on the surface but omit the procedural and temporal structure needed to understand the case as a legal dispute.

Fourth, numerically dense cases expose differences in both generation and evaluation. Monetary amounts, compensation breakdowns, transaction sequences, commissions, asset values, and medical or repair costs are easy to omit or compress during narrative synthesis. Evaluator models also differ in how strongly they penalize missing numerical details. As a result, these cases often produce divergent scores even when the general dispute structure is preserved.

These outliers show that failures are not simply model errors. They often arise from the interaction between model behavior and institutional data practices. De-identification, record compression, mixed reporting perspectives, and multi-case aggregation all shape whether a fact-construction workflow can operate reliably. The failure modes therefore point to a broader governance lesson: improving AI-assisted fact construction may require not only stronger models or better prompts, but also more structured role mapping, clearer separation of episodes, explicit flags for third-party reporting, and more systematic recording of numerical details. Additional illustrative outlier cases are provided in Appendix A.

## 5 Discussion

### 5.1 Fact construction as a distinct evaluation layer

The results support the central claim of this article: fact construction should be treated as a distinct layer in legal AI evaluation. Recent work on legal AI has increasingly moved beyond static question answering and classification benchmarks. Multi-agent debate frameworks, for example, have been used to improve legal judgment prediction by modeling adversarial or evaluative interaction (Chen et al., 2025). Dynamic legal environments have also been proposed to evaluate language agents across legal consultation, document drafting, and courtroom scenarios, emphasizing multi-role interaction, procedural constraints, and the difficulty of maintaining performance beyond static benchmarks (Jia et al., 2025). These studies mark an important shift from evaluating isolated model outputs toward evaluating agents within legally structured processes.

The present study builds on this shift but identifies a more specific and earlier layer of legal work. In many dynamic legal tasks, legally usable facts are often presumed to be available at the point where the model is asked to answer a legal question, draft a pleading, predict a judgment, or comply with procedural stages. Legal consultation, however, begins before such facts have been stabilized. Clients may not know which facts matter, may disclose information gradually, or may frame their situation through emotional, social, or procedural concerns rather than legal categories. If legal AI systems are evaluated only on downstream legal reasoning, drafting, or procedural performance, they may appear more reliable than they would be in real consultation settings, where factual insufficiency is itself part of the task.

The multi-agent workflow developed in this study makes this pre-reasoning layer visible. The Litigant Twin models constrained factual disclosure, the LegalAider models elicitation, the NarrativeAgent models synthesis, and the Evaluator models factual validation. This decomposition allows evaluation to ask not merely whether an LLM can produce a fluent legal answer or a procedurally compliant output, but whether the consultation process can elicit, preserve, and reconstruct a reliable factual narrative before legal analysis begins. In this sense, fact construction is not merely a component within legal consultation. It is a prior evaluation layer on which subsequent legal reasoning, drafting, and procedural performance depend.

### 5.2 From autonomous advice to supervised factual support

The study also clarifies how LLMs should be positioned in legal aid and clinical workflows. The findings do not support replacing lawyers, legal aid staff, or clinic supervisors with autonomous legal advice systems. The more appropriate use case is narrower: LLMs may assist early-stage intake, factual organization, and diagnostic review under professional supervision. In this setting, the system should not be understood as producing legal advice. It produces a provisional factual account that remains subject to human verification before any legal assessment, counseling, drafting, or strategic decision is made.

This distinction is important because the task examined in this study precedes doctrinal legal analysis. Its contribution lies in making factual materials easier to inspect: producing preliminary narratives, identifying missing information, preserving uncertainty, and highlighting possible inconsistencies. Used in this way, LLMs may reduce the cognitive burden of reviewing fragmented consultation materials without displacing the professional responsibility to decide which facts are legally relevant and how they should be evaluated.

For such support to be responsible, the output must remain contestable. Human reviewers should be able to trace the generated narrative back to the source record, inspect the dialogue through which facts were disclosed, identify unanswered or weakly supported points, and revise the factual account. The Evaluator's diagnostic comments can assist this review by flagging omissions, unsupported additions, contradictions, or uncertainty. They should not, however, be treated as authoritative determinations of factual truth.

This view treats human oversight as part of the fact-construction workflow rather than as an after-the-fact safety check. In legal aid and clinical settings, this is especially important because clients may be vulnerable, legally inexperienced, or unable to articulate their situation in legally relevant terms. A responsible LLM-supported workflow should therefore preserve the professional role of lawyers, supervisors, and trained students while using AI to make early-stage factual uncertainty more visible, structured, and reviewable.

### 5.3 Institutional data practices and AI reliability

A major finding of the outlier analysis is that institutional data practices shape the reliability of AI-assisted fact construction. The long-tail failures identified in this study were not caused only by model limitations. They also reflected how legal clinic records were anonymized, compressed, and organized. De-identification protects privacy, but it can obscure role relationships when multiple parties are replaced with abstract placeholders. Case records preserve institutional memory, but they may compress several incidents, proceedings, or transactions into a single factual account. Third-party reporting may be unavoidable in practice, but it creates uncertainty about who directly experienced the relevant events and which details remain second-hand.

These findings suggest that improving legal AI reliability does not always require a larger model or a more complex architecture. Some failures may be reduced through better record design. Legal clinics and legal aid organizations could maintain explicit role-mapping tables for anonymized records, separate multi-episode matters into distinct factual units, flag third-party reporting at intake, and record monetary amounts, dates, and procedural events in structured fields. These measures should not be understood as AI-specific requirements alone. They would also improve human supervision by making factual relationships, uncertainty, and missing information easier to review.

The broader implication is that responsible AI-assisted fact construction depends on the quality of the institutional record as well as the capability of the model. In legal consultation, factual reliability is jointly produced by clients, students, legal professionals, record-keeping practices, and computational tools. Treating institutional data practices as part of the AI workflow therefore helps shift attention from model performance alone to the socio-technical conditions under which factual narratives become reviewable and reliable.

### 5.4 Contestability and the limits of LLM-based evaluation

The moderate agreement among evaluator models reinforces the need for caution in LLM-based evaluation. Model-driven assessment is useful for scalable comparison, especially when the evaluation target is narrow and the criteria are explicit. In this study, however, evaluator scores are not treated as validated measurements of legal quality or factual truth. They are diagnostic signals produced under controlled conditions. Their value lies in revealing comparative patterns, unstable cases, and possible failure modes, not in replacing human expert judgment.

This limitation is especially important in legal domains because factual salience and legal relevance are often intertwined. A narrative may omit facts that appear minor at the surface level but become important once a legal issue is identified. Conversely, an evaluator may penalize missing details that do not materially affect the legally relevant factual account. For this reason, model-driven evaluation must remain contestable. Human reviewers should be able to inspect the source record, examine the generated narrative, question the assigned score, and determine whether the apparent mismatch reflects a generator failure, evaluator strictness, ambiguity in the source record, or evaluator error.

Future versions of this framework should therefore include human expert audit stratified across the score distribution. Such an audit would allow model-driven scores to be calibrated against external legal judgment and would help distinguish among genuinely incomplete narratives, strict but reasonable evaluator scoring, and evaluator error. Legal-domain precedents for GPT-assisted legal annotation suggest that model-based workflows become more credible when they are anchored in human validation rather than treated as autonomous evaluation systems (Gray et al., 2023).

### 5.5 Implications for access to justice and clinical legal education

For access-to-justice contexts, the value of AI-assisted fact construction lies in its potential to support the quality and efficiency of early-stage intake. The results suggest that, when consultation records are internally consistent, limited to a single dispute, and supported by clear actor-role relationships, the workflow can generate usable preliminary factual narratives for triage and review. Such support may be useful in resource-constrained legal aid organizations, where staff, students, and supervisors must process many consultations under time pressure. At the same time, the outlier analysis shows that this value is conditional. Cases involving third-party reporting, multi-episode histories, anonymized role confusion, or dense numerical details require more careful human review and more structured intake practices before AI-generated narratives can be relied upon.

For clinical legal education, the framework may serve as a training and reflection tool. Students can compare AI-generated narratives with original consultation records, identify missing questions, examine how uncertainty enters the factual account, and consider why some facts are preserved while others are omitted. The pedagogical value does not come from automating legal reasoning or replacing supervision. It comes from making fact elicitation and factual synthesis visible, reviewable, and open to critique. Used in this way, the framework can help students understand that legal facts are not simply collected; they are constructed through questioning, recording, interpretation, and review.

## 6 Limitations

This study has several limitations. First, the dataset consists of 100 de-identified records from a specific legal clinical context. The findings may not generalize to other jurisdictions, legal domains, or institutional settings. Second, the evaluation is based primarily on LLM-driven scoring. Although the evaluation target is narrow and the rubric is explicit, the scores have not yet been calibrated against systematic human expert judgments. This limits the strength of claims that can be made about measurement validity, especially at the level of individual cases. The scores should therefore be interpreted as comparative diagnostic signals rather than validated measures of factual alignment.

Third, the experiment does not include a direct single-agent summarization baseline. Such a baseline would allow stronger claims about the specific contribution of role separation. For this reason, the present results should be interpreted as a diagnostic analysis of the proposed role-separated workflow. Fourth, the study uses a single composite factual-alignment score guided by accuracy, completeness, and consistency, rather than independently validated sub-scores for each dimension. This design allows the evaluator to provide an overall diagnostic assessment of factual reconstruction, but it does not fully disentangle different types of factual-construction failure. Future work should collect and validate separate dimensional scores for accuracy, completeness, and consistency.

Fifth, the study evaluates factual adherence, not relevance filtering, legal issue spotting, doctrinal classification, legal advice quality, or outcome prediction. This limitation is also a design choice: the framework isolates fact construction as a prior layer of legal work before downstream legal analysis begins. Sixth, the original consultation records cannot be publicly released in full because they contain sensitive legal consultation material, even after de-identification. This limits full external replication using the same source records. Nevertheless, transparency and reproducibility are supported in the accompanying Supplementary material, which provides representative prompt templates for the LegalAider, Litigant Twin, NarrativeAgent, and Evaluator, together with the factual-alignment evaluation rubric and output schemas.

These limitations shape the interpretation of the results. The study should be read as a practice-grounded evaluation framework and diagnostic analysis, not as a definitive benchmark for legal consultation AI. Its contribution lies in making factual elicitation, narrative construction, evaluator disagreement, and recurring failure modes observable in a controlled workflow. A future fact-construction benchmark would require additional steps, including human expert calibration, inter-rater reliability analysis, baseline comparisons, and broader testing across legal domains and institutional settings.

## 7 Conclusion

This article argued that fact construction should be treated as a distinct evaluation layer in legal AI research. Legal AI evaluation often begins once facts have already been structured, but legal practice frequently requires facts to be elicited, clarified, stabilized, and narrated before legal reasoning can responsibly occur. Using a role-separated multi-agent workflow and 100 real-world legal consultation records, this study examined how LLM-based agents perform in this pre-reasoning layer. The aim was not to propose a definitive benchmark for legal consultation AI, but to develop a practice-grounded framework for making factual elicitation, narrative construction, and failure modes observable.

The empirical results show that LLM-based agents can generate usable factual narratives under constrained conditions, particularly when the source record is internally consistent, limited to a single dispute, and supported by clear actor-role relationships. At the same time, evaluator disagreement and outlier analysis reveal recurring sources of failure, including anonymized role confusion, third-party narration, multi-episode case complexity, and numerically dense information. These findings indicate that reliability in AI-assisted legal consultation depends not only on model capability, but also on interaction design, data representation, institutional record-keeping, and human oversight.

For responsible LLM integration in law, the implication is that legal AI systems should be evaluated not only for downstream reasoning performance, but also for their ability to support the construction of factual narratives in real socio-technical workflows. Fact construction is not a peripheral preprocessing step. It is a foundational layer of legal practice and a necessary condition for trustworthy, contestable, and accountable legal AI.

## Figures and Tables

**Table A1:** Illustrative cases of agency-level failure modes.

Case ID	Agent-generated content (abridged)	Human-written content (abridged)	Outlier category	Author briefing
105	Key facts largely unspecified; uncertainty regarding actors, events, and procedures.	Drug use incident leading to death with detailed timeline, caregiving payment, medical cause of death, and criminal prosecution.	Anonymization-induced referential ambiguity	The case record relied heavily on anonymized role labels and third-party narration by a friend rather than the primary actor. During interaction, the Litigant Twin repeatedly deferred responsibility and expressed uncertainty, preventing effective clarification.
121	General description of verbal harassment and emotional distress.	Multiple criminal incidents including assault, sexual violence, police misconduct, and data misuse across several years.	Multi-episode case compression	The underlying case consists of multiple legally distinct events unfolding over several years. The agent collapsed these heterogeneous episodes into a single narrative, omitting procedural history and legal escalation.
136	Vague account of possible online fraud involving a foreign student.	Money transfers, gaming point purchases, and prosecution for money laundering.	Third-party reporting limitation	The inquirer was not the primary party to the transaction but reported events concerning a partner. The Litigant Twin consistently avoided committing to factual details, leading the agent to over-report uncertainty.
040	Abstract family conflict without contractual or financial specificity.	Inheritance agreements, asset values, statutory claims, and litigation history.	Anonymization-induced referential ambiguity	Coded identifiers for multiple family members disrupted role consistency. The LegalAider could not reliably map anonymized labels back to real persons, resulting in ineffective fact elicitation.
164	Suspected fraud with unexplained account freezing.	Three-party fraud scheme with transaction amounts, commissions, and police reports.	Incomplete self-disclosure by the Litigant Twin	The human-written record indicates participation in purchasing prepaid cards, but the Litigant Twin initially denied entering into any transaction, creating a narrative gap.
079	Single altercation described without procedural follow-up.	Police intervention, medical reports, prosecution, and counterclaims across multiple dates.	Multi-episode case compression	The case involved repeated incidents, medical treatment, and criminal proceedings, but the agent focused narrowly on the initial altercation.
113	Inheritance uncertainty without concrete figures or dates.	Detailed land valuations, withdrawals, and contested distribution agreements.	Incomplete self-disclosure by the Litigant Twin	Despite access to documentary records, the Litigant Twin repeatedly withheld precise financial amounts and dates, resulting in an under-specified narrative.
144	Partial family roles described; legal dispute unclear.	Inheritance manipulation involving agents, omitted heirs, and prolonged litigation.	Multi-episode case compression	The agent followed the prompt structure too rigidly and failed to probe the alleged fraudulent intent behind intermediary actions.
163	Traffic accident summarized with general liability uncertainty.	Precise compensation breakdown and negotiation positions documented.	Incomplete self-disclosure by the Litigant Twin	Factual events were largely consistent, but numerical details regarding damages and compensation were unevenly disclosed. Evaluator disagreement reflects sensitivity to quantitative completeness.

## Data Availability

Access to the underlying legal consultation records is restricted due to confidentiality, privacy protection, and institutional data governance requirements. These records are not publicly available. Requests for further information regarding data access may be directed to the corresponding author.

## Appendix A. Illustrative outlier cases

The following cases provide illustrative examples of the failure modes identified in the main analysis. They are presented as qualitative evidence of how misalignment between agent roles, source records, and interaction structure manifests in practice.
