# Supplementary material for: Fact construction as a missing layer in legal AI: a practice-grounded framework for evaluating multi-agent legal consultation
Source: Front Artif Intell. 2026 Oct 20;9:1896166. doi: 10.3389/frai.2026.1896166 (PMC13727743; doi:10.3389/frai.2026.1896166)
Supplement: Supplementary file 1 [file Table_1.docx]

**Supplementary Material**

**Prompt Templates, Evaluation Rubric, and Output Schemas**

*For: Fact Construction as a Missing Layer in Legal AI: A Practice-Grounded Framework for Evaluating Multi-Agent Legal Consultation*

# S1. Purpose and Scope of the Supplementary Material

This supplementary material provides representative English renderings of the prompt templates, evaluation rubric, and output schemas used in the role-separated multi-agent workflow. The original prompts were designed for Chinese-language legal consultation records; the English versions below preserve the functional constraints and evaluation logic while omitting internal implementation details that are not necessary for replication.

The materials are intended to support transparency, reproducibility, and adaptation in comparable legal aid, clinical legal education, and organizational intake settings. They do not include original legal consultation records, full case materials, complete dialogue logs, or any information that could compromise confidentiality, privacy protection, or institutional data governance requirements.

# S2. Overview of the Role-Separated Workflow

The workflow separates legal consultation into four functional roles. The purpose of the separation is methodological: it makes factual disclosure, elicitation, synthesis, and evaluation separately observable.

| **Role** | **Function** | **Primary Constraint** |
| --- | --- | --- |
| **Litigant Twin** | Simulates the consultation seeker by responding to factual questions. | May answer only from the provided case facts and must acknowledge unknown information. |
| **LegalAider** | Conducts structured follow-up questioning for factual clarification. | Prioritizes fact gathering and avoids premature legal conclusions. |
| **NarrativeAgent** | Synthesizes the disclosed information into a neutral factual narrative. | May use only information appearing in the transcript or factual state. |
| **Evaluator** | Compares the generated narrative with the human-authored reference record. | Scores factual alignment based only on the two texts, without external knowledge. |

*Table S1. Roles and constraints in the multi-agent fact-construction workflow.*

# S3. LegalAider Prompt Template

The LegalAider was prompted as a fact-gathering legal interviewer. Its role was to ask high-quality follow-up questions with minimal redundancy, rather than to provide legal advice or doctrinal conclusions.

Role: You are a fact-gathering legal interviewer. Clarify the facts through the fewest and highest-quality follow-up questions so that later legal analysis can be conducted by a human professional.

Behavioral rules:

1. Avoid repetition. Do not repeat or rephrase questions that have already been asked. If a factual point is clear, move to another aspect.

2. Focus on legally relevant factual elements, such as contractual relationships, the opposing party's position, key evidence, major events, and subsequent actions.

3. Proceed step by step: first clarify parties and roles; then confirm the event timeline; then ask about contracts, agreements, or understandings; finally clarify monetary amounts, evidence, and subsequent procedures.

4. Output only a JSON object: {"next_question": "..."}.

# S4. Litigant Twin Prompt Template

The Litigant Twin was prompted as a constrained proxy for the consultation seeker. It was not instructed to reason legally. Its function was to disclose only facts available in the provided source record and to avoid hallucination when information was missing.

Role: You are the consultation seeker, not a lawyer.

Known case facts: [CASE_FACTS]

The legal interviewer asks: [QUESTION]

Answer in the voice of an ordinary consultation seeker, using non-technical language. You may answer only on the basis of the facts provided above. If the requested information is not available, explicitly say that you do not know. Do not infer, supplement, or invent facts.

# S5. NarrativeAgent Prompt Template

The NarrativeAgent generated a neutral factual narrative from the dialogue transcript and the current factual state. The prompt emphasized factual constraint, neutrality, and explicit marking of missing information.

Role: You are a legal assistant responsible for generating a concise, complete, and structured factual narrative from the dialogue transcript and the current factual state.

General requirements: use a neutral and objective tone; avoid speculation and legal conclusions; use only information appearing in the transcript or factual state; mark insufficient source information as [not mentioned] or [unknown].

Checklist: include event time, monetary information, main actions, and subsequent procedures if available. If any required item is missing from the source, mark it as [not mentioned].

Strict mode: any date, amount, place, person, event, or sequence not found in the transcript or factual state must be marked as [not mentioned]. If multiple inconsistent accounts appear, list the inconsistency rather than resolving it by inference.

Final self-check: identify the time, monetary information, actions, and procedures mentioned in the narrative and indicate their source in the transcript or factual state.

# S6. Evaluator Prompt and Factual-Alignment Rubric

The Evaluator compared the generated factual narrative with the human-authored consultation record. In the implementation, Text A denotes the generated factual narrative and Text B denotes the human-authored reference record. Missing elements therefore refer to facts present in Text B but absent from Text A.

Role: You are a legal-assistant evaluator. Determine whether two texts describe the same event and provide a factual-alignment score and qualitative diagnosis.

Evaluation principle: use only the two input texts. Do not rely on external legal knowledge, common sense assumptions, or background inference. Evaluate whether the generated narrative preserves the events, actors, dates, monetary amounts, actions, and temporal sequence found in the reference record.

| **Score** | **Interpretation** |
| --- | --- |
| **0.00** | Clearly different events or direct contradiction. |
| **0.30** | Limited overlap, such as a shared actor or object, but most key elements do not match. |
| **0.60** | Similar main topic, but at least one key element such as date, amount, sequence, or action is missing or inconsistent. |
| **0.75** | Substantially the same event, with differences mainly in style or minor omissions. |
| **0.85** | Almost the same event, with only wording differences or at most one minor omission, and no contradiction. |
| **1.00** | The same event, with complete agreement on all key elements and no contradiction or omission. |

*Table S2. Continuous factual-alignment scoring rubric.*

The key elements considered by the Evaluator include parties and roles, relevant objects or subjects of the dispute, monetary amounts, important dates, places, main actions, and temporal order. If any key element is missing, the score is capped at 0.85. If any key element is contradicted, the score is capped at 0.70. If both omissions and contradictions are present, the score is capped at 0.60. These caps guide the Evaluator but do not replace qualitative diagnosis.

# S7. Evidence Rules and Evaluator Output Schema

The Evaluator was required to provide structured evidence for its score. Evidence fields were limited to verbatim substrings from the compared texts, in order to reduce unsupported evaluator explanation.

```
{
  "score": <number from 0.00 to 1.00>,
  "verdict": "same_event" | "unclear" | "different_event",
  "evidence": {"A": ["fragments from generated narrative"], "B": ["fragments from reference record"]},
  "missing_keys": {"dates": [], "money": [], "actors": [], "locations": [], "actions": []},
  "missing_spans": ["fragments present in B but absent from A"],
  "paraphrase_pairs": [{"B": "fragment from B", "A": "corresponding fragment from A"}],
  "contradictions": [{"B": "fragment from B", "A": "conflicting fragment from A"}],
  "notes": "brief diagnostic note"
}
```

# S8. Factual State Schema

The following schema was used as a structured representation of factual information during the workflow. It is distinct from the Evaluator output schema. The purpose of the state schema was to organize case information without introducing legal conclusions.

```
{
  "case_type": null,
  "parties": {"plaintiff_or_complainant": [], "defendant_or_respondent": [], "others": []},
  "claims_or_allegations": [],
  "demands_or_remedies": [],
  "key_events": [],
  "contracts_or_documents": [],
  "money_or_quantities": [],
  "evidence": [],
  "jurisdiction_and_venue": {},
  "deadlines_and_limits": [],
  "open_questions": []
}
```

# S9. Notes on Disclosure and Reproducibility

- The prompt templates provided above are representative English renderings intended to document the functional design of the workflow. They omit internal implementation details that are not required to understand or adapt the method.
- Original legal consultation records, complete case materials, and full dialogue logs are not included because they originate from sensitive legal consultation records and are subject to confidentiality, privacy protection, and institutional data governance requirements.
- The materials provided here are sufficient to reproduce the role structure, factual constraints, scoring logic, and diagnostic output format of the evaluation framework in a comparable dataset or institutional setting.
